# Relationship Between Cortical Gyrification, White Matter Connectivity, and Autism Spectrum Disorder

**DOI:** 10.1093/cercor/bhw098

**Published:** 2016-04-29

**Authors:** C. Ecker, D. Andrews, F. Dell'Acqua, E. Daly, C. Murphy, M. Catani, M. Thiebaut de Schotten, S. Baron-Cohen, M.C. Lai, M.V. Lombardo, E.T. Bullmore, J. Suckling, S. Williams, D.K. Jones, A. Chiocchetti, the MRC AIMS Consortium, D.G.M. Murphy

**Affiliations:** 1 Department of Forensic and Neurodevelopmental Sciences, and the Sackler Institute for Translational Neurodevelopmental Sciences, Institute of Psychiatry, Psychology and Neuroscience, King's College, London SE5 8AF, UK; 2 Department of Child and Adolescent Psychiatry, Psychosomatics and Psychotherapy, Goethe University, 60528 Frankfurt am Main, Germany; 3 Autism Research Centre, Department of Psychiatry, University of Cambridge, Cambridge CB2 8AH, UK; 4 Child and Youth Mental Health Collaborative at the Centre or Addiction and Mental Health and The Hospital for Sick Children, Department of Psychiatry, University of Toronto, Toronto, Ontario, M6J 1H4, Canada; 5 Department of Psychiatry, National Taiwan University Hospital and College of Medicine, Taipei, Taiwan 100, R.O.C; 6 Department of Psychology and Center for Applied Neuroscience, University of Cyprus, 1678 Nicosia, Cyprus; 7 Brain Mapping Unit, Department of Psychiatry, University of Cambridge, Cambridge CB2 0SZ, UK; 8 Centre for Neuroimaging Sciences, Institute of Psychiatry, Psychology and Neuroscience, King's College, London SE5 8AF, UK; 9 Cardiff University Brain Research Imaging Centre, School of Psychology, Cardiff University, Cardiff CF24 5HQ, UK

**Keywords:** Autism spectrum disorder, brain anatomy, brain connectivity, brain development, multimodal neuroimaging

## Abstract

Autism spectrum disorder (ASD) is a complex neurodevelopmental condition, which is accompanied by differences in gray matter neuroanatomy and white matter connectivity. However, it is unknown whether these differences are linked or reflect independent aetiologies. Using a multimodal neuroimaging approach, we therefore examined 51 male adults with ASD and 48 neurotypical controls to investigate the relationship between gray matter local gyrification (*l*GI) and white matter diffusivity in associated fiber tracts. First, ASD individuals had a significant increase in gyrification around the left pre- and post-central gyrus. Second, white matter fiber tracts originating and/or terminating in the cluster of increased *l*GI had a significant increase in axial diffusivity. This increase in diffusivity was predominantly observed in tracts in close proximity to the cortical sheet. Last, we demonstrate that the increase in *l*GI was significantly correlated with increased diffusivity of short tracts. This relationship was not significantly modulated by a main effect of group (i.e., ASD), which was more closely associated with gray matter gyrification than white matter diffusivity. Our findings suggest that differences in gray matter neuroanatomy and white matter connectivity are closely linked, and may reflect common rather than distinct aetiological pathways.

## Introduction

Autism spectrum disorder (ASD) comprises a group of heterogeneous neurodevelopmental conditions characterized by impaired communication, social reciprocity, and repetitive/stereotypic behavior (Wing 1997). Despite the large degree of genetic and phenotypic heterogeneity, several lines of evidence now converge in suggesting a common etiological pathway for ASD, that is, defective synaptic structure and aberrant brain connectivity (Betancur et al. 2009). However, the notion of atypical brain connectivity in ASD is complex, involving abnormalities within the gray and white matter (Ecker et al. 2013; Ameis and Catani 2015), and different levels of integration (Belmonte et al. 2004). To fully characterize the neural systems underlying ASD, it is thus important to explore different aspects of brain anatomy and connectivity within a unitary framework.

So far, evidence for altered structural brain connectivity in ASD mainly comes from neuroimaging studies investigating white matter (WM). For example, voxel-based morphometry studies reported that individuals with ASD have spatially distributed reductions in regional WM volume during childhood (McAlonan et al. 2005), adolescence (Waiter et al. 2005), and adulthood (Ecker et al. 2012). Atypical structural connectivity in ASD has also been noted in numerous diffusion tensor imaging (DTI) studies, particularly in fiber-tracts mediating autistic symptoms and traits (e.g., language and limbic pathways (Pugliese et al. 2009), fronto-striatal and fronto-occipital circuitry (Sahyoun et al. 2010; Langen et al. 2012; Catani et al. 2016), corpus callosum (Alexander et al. 2007), hippocampo-fusiform and amygdalo-fusiform pathways (Conturo et al. 2008), and the cerebellar circuitry (Catani et al. 2008)) (for review, see Ameis and Catani 2015). Taken together, these studies support the hypothesis that ASD is a “neurodevelopmental disconnection syndrome” associated with the altered formation of fiber pathways (Courchesne and Pierce 2005; Geschwind and Levitt 2007; Wolff et al. 2012), which accompanies the neurodevelopmental differences in gray matter (GM) that are typically observed in individuals with ASD (for review, see Amaral et al. 2008; Ecker et al. 2015).

Most prior neuroimaging studies examine differences in GM or WM in isolation, therefore the relationship between atypical GM and WM in ASD therefore remains poorly understood. Studies examining typical brain development suggest that the formation of the WM neurocircuitry is intrinsically linked to developmental mechanisms underlying GM maturation. For example, the formation of principal axonal and dendritic projections—and subsequent myelination—builds upon the completion of neuronal proliferation, migration, and differentiation (Kostovic and Rakic 1980; Tau and Peterson 2010; Molnár et al. 2014). If perturbed, as has been suggested in ASD (Abrahams and Geschwind 2010; Pinto et al. 2010; Parikshak et al. 2013), the mechanisms that drive atypical GM development are thus likely to also interfere with the development of WM. Moreover, genetic studies link ASD to developmental events affecting axonal growth and guidance (e.g., Pinto et al. 2014), which may affect the formation of the WM circuitry in addition to perturbed GM development. In order to elucidate the causal mechanisms underlying the cortical systems pathology of ASD, it is important to determine whether variations in GM neuroanatomy and WM connectivity are linked, or are independently modulated by ASD.

While longitudinal studies during early brain development would be required to establish a causal link between GM and WM abnormalities in ASD, a recent cross-sectional study suggests that altered WM connectivity in children with ASD is significantly associated with atypical GM gyrification (Schaer et al. 2013). However, this association was only observed in intra-lobar, but not in inter-hemispheric connections, suggesting that the relationship between GM and WM may be scale-dependent, with short-distance tracts being more closely associated with GM variations than long-distance tracts. This finding extends a previous neuroimaging study showing that the nature of volumetric WM differences in ASD may be related to their proximity to the cortical sheet (Herbert et al. 2004), with tracts in close proximity to the cortical sheet potentially being more affected than tracts deep within the cortical WM. Last, there is histological evidence to suggest that WM differences in ASD are dependent on the length of axonal projections, and is characterized by a decrease in the number of large axons communicating over long distances, but an excessive number of thin axons linking neighboring areas (Zikopoulos and Barbas 2010). Taken together, these findings provide a mechanism for the disconnection of long-distance pathways, and excessive short-distance connections in ASD (Belmonte et al. 2004; Courchesne and Pierce 2005; Casanova et al. 2006).

Here, using a combined structural magnetic resonance imaging (MRI) and DTI approach, we examined the relationship between GM neuroanatomy and WM connectivity in male adults with ASD and typically developing (TD) controls. As in Schaer et al. (2013), we investigated GM anatomy based on measures of surface area and local gyrification, as the degree of cortical gyrification has previously been linked to various different aspects of the cortical architecture (e.g., cellular complexity, neuronal density, cellular alignment; Welker 1990) that are expected to alter the underlying WM connectivity demands (Casanova 2004). However, unlike Schaer et al. (2013), we initiated tractography from surface-based clusters with significant between-group differences in cortical folding, and also separated short from long tracts based on their respective distance from the cortical sheet. It was hypothesized that measures of GM gyrification and WM connectivity are significantly linked, and are thus likely to represent a common etiological pathway in ASD.

## Materials and Methods

### Participants

Fifty-one male right-handed adults with ASD and 48 typically developing (TD) male controls aged 18–43 years were recruited by advertisement and assessed at the Institute of Psychiatry, Psychology and Neuroscience (IoPPN), London, and the Autism Research Centre, University of Cambridge, UK. Approximately equal ratios of cases to controls were recruited at each site: London 27:24 (ASD:TD) and Cambridge (23:25). Groups were matched for age, full-scale IQ, ethnicity, and handedness. All participants with ASD were diagnosed during adulthood according to ICD-10 research criteria and using the Autism Diagnostic Interview-Revised (ADI-R; Lord et al. 1994). All cases reached algorithm cut-offs in the 3 domains of the ADI-R, although failure to reach cut-off in one domain by one point was permitted. Current symptoms were assessed using the Autism Diagnostic Observation Schedule (ADOS; Lord et al. 1989). ADI-R rather than ADOS scores were chosen as exclusion criteria as current symptoms during adulthood are often masked by coping strategies and can also be alleviated by treatment/intervention. The ADI-R diagnosis thus ensured that all individuals with ASD met the criterion of childhood autism. Further exclusion criteria included contraindication to MRI, a history of major psychiatric disorder (e.g., psychosis), head injury, genetic disorder associated with autism (e.g., fragile-X syndrome, tuberous sclerosis), or any other medical condition affecting brain function (e.g., epilepsy). We also excluded participants on antipsychotic medication, mood stabilizers, or benzodiazepines. Overall intellectual ability was assessed using the Wechsler Abbreviated Scale of Intelligence (WASI; Wechsler 1999). All participants fell within the high-functioning range on the spectrum defined by a Full Scale IQ (FSIQ) >70. All participants gave informed written consent in accordance with ethics approval by the National Research Ethics Committee, Suffolk, UK.

### Structural MRI and DTI Data Acquisition

Scanning took place at the IoPPN, London, and the Addenbrooke's Hospital, Cambridge, using a 3T GE HDx Signa System (General-Electric, Milwaukee, USA). Details of the acquisition protocol have been described elsewhere (Deoni et al. 2008; Ecker et al. 2012). Initially, multisite compatible quantitative *T*_1_-maps were used to derive high-resolution structural *T*_1_-weighted inversion-recovery images, with 1 × 1 × 1 mm resolution, a 256 × 256 × 176 matrix, repetition time (TR) = 1800 ms, time to inversion = 50 ms, fractional anisotropy (FA) = 20°, and field of view (FOV) = 25 cm. Subsequently, DTI data using a spin-echo echo-planar imaging double refocused sequence providing whole-head coverage with isotropic image resolution (2.4 × 2.4 × 2.4 mm) were acquired (32 diffusion-weighted volumes with different noncollinear diffusion directions (Jones et al. 1999) with *b*-value 1300 s/mm^2^ and 6 nondiffusion-weighted volumes; 60 slices; no slice gap; time echo = 104.5 ms; TR = 20 R–R interval; 128 × 128 acquisition matrix; FOV 307 × 307 mm). The acquisition was peripherally gated to the cardiac cycle.

### Cortical Reconstruction Using FreeSurfer

The FreeSurfer software package (http://surfer.nmr.mgh.harvard.edu/) was used to derive models of the cortical surface for each *T*_1_-weighted image. These well-validated and fully automated procedures have been extensively described elsewhere (e.g., Dale et al. 1999). The resulting surface models were visually inspected for reconstruction errors, and surface reconstructions with visible inaccuracies were further excluded from the statistical analysis (dropout < 10%) prior to sample generation.

Vertex-level measures of gyrification were derived as described by Schaer et al. (2008). The local gyrification index (*l*GI) is a local variant of the classical 2-dimensional (2D) gyrification index originally proposed by Zilles et al. (1988), which is defined as the ratio of the total pial surface area over the perimeter of the brain delineated on 2D coronal sections (Zilles et al. 1988). The *l*GI utilizes the high-resolution surface reconstructions provided by FreeSurfer to measure the degree of gyrification at each cerebral vertex, thus providing 3D measures of local gyrification at each spatial location on the entire cortical surface. The *l*GI at a given vertex *v_i_* is computed as the ratio between the surface of a circular patch (i.e., geodesic circle with radius *r* centered at *v_i_*) on the outer surface of the brain, and the surface of the corresponding patch at *v_i_* on the pial surface (vertex positions are preserved across surfaces)*.* Thus, the *l*GI at each point *v_i_* reflects the amount of cortex buried within the sulcal folds in the surrounding area (Schaer et al. 2008). Clusters of significant between-group differences in *l*GI on the cortical surface were subsequently converted to 3D regions of interest (ROIs) for automated tract dissections. We also examined between-group differences in vertex-wise estimates of cortical surface area (SA), which were derived as outlined previously (Winkler et al. 2012). Last, we extracted measures of mean cortical thickness (mean CT), sulcal depth, and mean (i.e., radial) curvature from the cluster of significant between-group difference in *l*GI in order to test whether differences in *l*GI are driven by other morphometric measures.

### DTI Preprocessing

Diffusion data were preprocessed and analyzed using ExploreDTI software (Leemans et al. 2009), as described elsewhere (Langen et al. 2012). Initially, whole-brain tractography was performed using all voxels with a FA ≥ 0.2. Seed-points were sampled from a uniform grid at the same resolution of the diffusion dataset. Streamlines were propagated using an Euler integration applying a cubic-spline interpolation of the diffusion tensor field, and with a step-size of 1 mm. Where FA < 0.2 or when the angle between 2 consecutive tractography steps was >35°, tractography stopped. A minimum length threshold of 20 mm was also applied to exclude ultra-short and spurious streamlines. Finally, diffusion tensor maps and whole-brain tractography were exported to TrackVis (Wang et al. 2007) for ROI-based tract dissection and visualization.

### Automated Tract Dissections Using Surface-based ROI(s)

**Figure 1.**
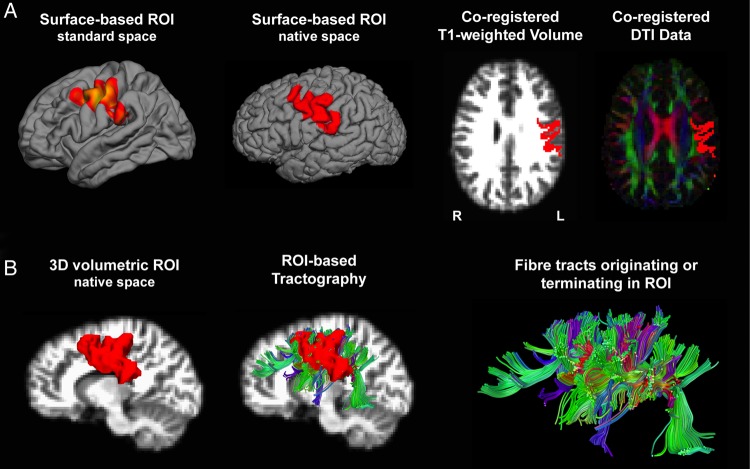
Automated tract dissections using surface-based clusters. (*A*) The cluster of significant between-group difference in *l*GI was initially mapped from the average surface in standard space to each individual's reconstructed surface in native space. For each individual, the surface-labels were then coregistered with the individual's structural volume and DTI data to create 3D volumetric ROIs. (*B*) The volumetric ROIs were subsequently used for automated fiber tracking, dissecting all tracts originating and/or terminating in the surface-based cluster. These tracts mainly included the arcuate fasciculus, the frontal inferior longitudinal fasciculus, ascending and descending projection tracts and local U-shaped fibers.

To examine the relationship between variations in *l*GI and characteristics of the underlying white-matter connections, the individual's structural MRI data (*T*_1_-weighted volumes and brain surfaces) were firstly coregistered with the DTI data (FA map) using FreeSurfer tools and FSL FLIRT (fsl.fmrib.ox.ac.uk/fsl/fslwiki/FLIRT). To create volumetric ROIs, the surface cluster(s) with a significant between-group difference in *l*GI were then mapped from the average standard-space cortical surface to the individual's reconstructed WM surface in native space, thus preserving the individuals' pattern of cortical folding. The surface-based clusters in native space were then converted to 3D volumes, which were subsequently used as “seed” regions to dissect out tracts originating and/or terminating in the surface-based ROI (i.e., we excluded tracts only passing through these ROIs) (Fig. 1). The MATLAB toolbox (The Mathworks, MA) “along-tract statistics” (Colby et al. 2012) was used to measure mean FA, mean diffusivity (MD), axial (perpendicular) diffusivity (AD), and radial diffusivity (RD) across dissected streamlines for each participant, in addition to calculations of the overall number of streamlines.

In order to generate a control region of comparable size and underlying tract architecture, the cluster(s) of significant differences in *l*GI were mapped to homolog regions in the contralateral hemisphere via a symmetric template (Greve et al. 2013) (see Supplementary Fig. 1), and tract dissections were performed as outlined above.

### Distribution of Tract Lengths

Prior to the statistical analysis of tract-specific DTI measures, the statistical distribution of tract lengths across subjects was examined to separate short- from long-distance tracts based on their respective lengths. Initially, the Hartigan's dip statistic (Hartigan and Hartigan 1985) was used to test for multimodality in the distribution, where a significant deviation from uni-modality is assumed to be indicative of multiple tract classes based on their length. Subsequently, Gaussian Mixture Models (EM algorithm in R for statistical computing) were used to model the distribution (and length cut-offs) for individual classes. The EM algorithm returns the probability density function *p* of *k* Gaussian mixture components, with

$$P(x)=\overset{k}{\underset{i=1}{\sum }}\lambda_{i}n_{i}(x;\mu ,\sigma ),$$

where *n_i_(x; μ, σ*) represents the *k*th Gaussian probability density function with mean *μ* and standard deviation *σ*. The number of components *k* was determined by means of cross-validation, which estimates the log-likelihood for different component solutions by performing a simple dataset splitting, where a randomly selected half of the data is use to fit the model, and half to test. Conventionally, a likelihood ratio test is performed to compare the goodness of fit of 2 (or more) models with different model parameters. Here, we simply chose the model with the fewest number of *n* Gaussian components that provided a considerable increase in log-likelihood relative to the *n* + *1* component model. Depending on the number of components and their respective length cut-offs, streamlines were separated into short- and long-distance tract classes based on their proximity to the surface-based label.

### Statistical Comparison

#### Vertex-wise between-group Differences in lGI and SA

Exploratory vertex-based statistical analysis of *l*GI and SA measures was conducted using the SurfStat Toolbox (www.math.mcgill.ca/keith/surfstat/) for MATLAB. To improve the ability to detect population changes, the *l*GI and SA maps were smoothed with a 5-mm full-width at half-maximum surface-based Gaussian kernel. Parameter estimates for vertex-wise *l*GI and SA estimates (*Y_i_*) were estimated by regression of a general linear model (GLM) at each vertex *i* with diagnostic group, and center as categorical fixed-effects factor, and age and FSIQ as continuous covariates:

$$Y_{i}=\beta_{0}+\beta_{1}\;\text{Group}+\beta_{2}\;\text{Center}+\beta_{3}\;\text{Age}+\beta_{4}\;\text{FSIQ}+\epsilon_{i},$$

where *ϵ_i_* is the residual error. Between-group differences were estimated from the coefficient *β_1_* normalized by the corresponding standard error. Corrections for multiple comparisons across the whole brain were performed using “random field theory” (RFT)-based cluster analysis for nonisotropic images using a *P* < 0.05 (2-tailed) cluster-significance threshold (Worsley et al. 1999). We also examined whether inter-individual variability in total brain measures (total brain volume, total surface area) affected the between-group difference in *l*GI, and in the subsequently described DTI measures, by including total brain volume as continuous covariate.

#### Between-group Differences in DTI Measures Using Surface-based ROIs

Statistical analysis of DTI measures in fiber tracts originating or terminating in the surface-based ROI was conducted to (1) examine between-group differences in white-matter microstructure in the ROI, and (2) to establish the relationship between measures of gyrification and underlying white matter diffusion properties. Here, a multivariate GLM (*R* Software, http://www.r-project.org/) was initially used to examine between-group differences in tract-specific DTI measures including diagnostic group and center as categorical fixed effects, and age and FSIQ as continuous covariates, using an initial test-wise error rate of *P* < 0.05 (2-tailed). Experiment-wide false positives were controlled for using Bonferroni corrections based on the number of independent comparisons conducted, resulting in a corrected test-wise error rate of *P* < 0.00625 (*n* = 8; see Table 2).

#### Relationship Between lGI and DTI Measures

Univariate GLMs were used to examine the relationship between local gyrification, white matter tract characteristics, and diagnostic status in clusters with significant between-group differences in *l*GI. Initially, we examined the effects of diagnostic status and local gyrification on diffusion properties of tracts with a significant between-group difference, while covarying for potential confounds of multisite acquisition (i.e., DTI*_i_* = *β*_0_ + *β*_1_Group + *β*_2_*l*GI + *β*_3_[Group *× l*GI] + *β*_5_Center + *ϵ_i_*). Analyses were performed (1) for all tracts originating and/or terminating in the surface-based ROI, and (2) for short- and long-distance tracts separately. Subsequently, we also examined the effect of variability in diffusion measures and diagnostic status on the degree of local gyrification (i.e., *l*GI*_i_* = *β*_0_ + *β*_1_ Group + *β*_2_DTI + *β*_3_[Group × DTI] + *β*_4_Centre + *ϵ_i_*).

Seemingly unrelated regression equations (SURE) (Zellner and Ando 2010) as implemented in the *R* software package were used to examine (1) whether the effect of group on measures of *l*GI was significantly larger than the effect of group on measures of diffusion; and (2) whether the effect of *l*GI on measures of diffusion significantly exceeded the effect of diffusion properties on *l*GI. Here, a *χ*^2^ test (*P* < 0.05) was used to compare a model in which parameters of interest were fixed across equations with a model in which parameters of interest were variable across equations.

Last, to identify the degree to which the relationship between the *l*GI and diffusion properties is significantly modulated by other cortical features, we examined partial correlation coefficients (*r*_par_) between the *l*GI and diffusivity measures while covarying for variability in mean CT, sulcal depth, and mean curvature within significant clusters. Furthermore, we examined whether these additional features are predictive of WM characteristics in addition to the *l*GI.

## Results

### Participant Demographics

There were no significant differences between individuals with ASD and TD controls in age (*t*_(97)_ = 1.04, *P* = 0.299) or full-scale IQ (*t*_(97)_ = 1.03, *P* = 0.305). There were also no significant between-group differences in total surface area (*t*_(97)_ = −0.613, *P* = 0.541), total GM volume (*t*_(97)_ = −0.89, *P* = 0.382), total WM volume (*t*_(97)_ = 0.964, *P* = 0.337), or total brain volume (*t*_(97)_ = 0.128, *P* = 0.899). In individuals with ASD, there was a significant increase in the ratio of total gray-to-white matter volume relative to controls (*t*_(97)_ = 2.831, *P* = 0.006) (Table 1).

**Table 1:** Subjects demographics and global brain measures

	ASD (*n* = 51)	TD controls (*n* = 48)
Age, years	26 ± 7 (18–43)	28 ± 6 (18–43)
Full-scale IQ (WASI)	112 ± 13 (77–135)	115 ± 10 (86–137)
ADI total	35 ± 9 (21–57)	–
ADI-R social	18 ± 5 (9–28)	–
ADI-R communication	14 ± 4 (8–24)	–
ADI-R repetitive behaviour	5 ± 2 (2–10)	–
ADOS total	10 ± 4 (1–21)	–
Total surface area (cm^2^)	2523 ± 252	2494 ± 215
Total white matter volume (cm^3^)	488 ± 58	499 ± 53
Total gray matter volume (cm^3^)	763 ± 86	749 ± 72
Total brain volume (cm^3^)	1251 ± 136	1248 ± 118
Ratio total gray:white matter	1.568 ± 0.017	1.505 ± 0.099*

Note: Data expressed as mean ± standard deviation (range), surface area expressed as cm^2^. There were no significant between-group differences in age, FSIQ, or global brain measures at *P* < 0.05 (2-tailed). *There was a significant between-group difference in the ratio of total GM to total WM (*P* < 0.05).

### Between-group Differences in lGI

**Figure 2.**
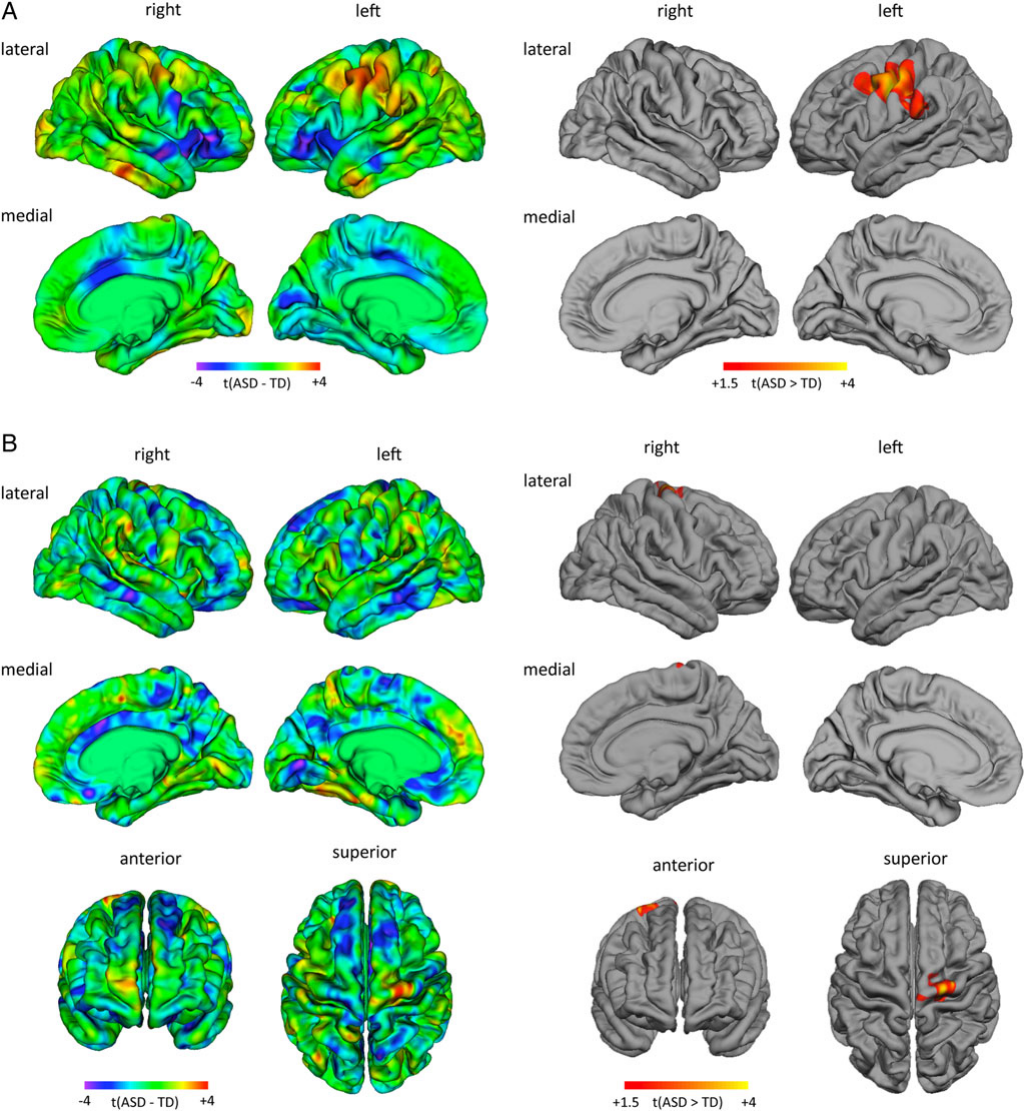
Between-group differences in *l*GI (*A*) and vertex-wise estimates of surface area (*B*). The left panel shows the un-thresholded *t*-maps where increased parameter estimates in ASD relative to controls are indicated in yellow-red, and decreases in cyan-blue. The right panel shows the random-field theory-based cluster-corrected (*P* < 0.05) difference map indicating clusters of significantly increased *l*GI and surface area in ASD relative to neurotypical controls. There were no significant clusters where individuals with ASD had significantly reduced *l*GI or surface area.

Relative to controls, individuals with ASD had significantly increased local gyrification in a large left-hemisphere cluster (*t*_max_ = 3.59, *n*_vertices_ = 9341, *P*_cluster_ = 0.026) centered around the central sulcus (Talairach *x* = −47, *y* = −7, *z* = 32), which included the primary motor and pre-motor cortex of the pre-central gyrus, and a small portion of the posterior middle frontal gyrus (approximate Brodmann area(s) [BA] 4/6), the somatosensory cortex on the post-central gyrus (approximate BA 3/1/2), and part of the supramarginal gyrus (BA 40) in the inferior parietal lobule. There were no regions in which individuals with ASD had significantly reduced *l*GI relative to controls (Fig. 2*A*). The same cluster of increased *l*GI in ASD was also observed when covarying for total brain volume or total surface area (see Supplementary Fig. 2). Within the cluster of increase *l*GI, there were also no significant between-group differences in mean CT (*t*_(97)_ = 1.51, *P* = 0.133), sulcal depth (*t*_(97)_ = −1.57, *P* = 0.119), or radial curvature (*t*_(97)_ = −1.69, *P* = 0.093).

### Between-group Differences in Surface Area

Individuals with ASD had significantly increased surface area as compared with controls in a small right-hemisphere cluster (*t*_max_ = 3.59, *n*_vertices_ = 1739, *P*_cluster_ = 0.017) centered on the superior precentral gyrus (i.e., primary motor cortex) (Talairach *x* = 23, *y* = −16, *z* = 65). There were no regions in which individuals with ASD had significantly reduced surface area relative to controls (Fig. 2*B*).

### Distribution of Tract Lengths

The left-hemisphere cluster of significant between-group differences in *l*GI provided a 3-dimensional ROI for each subject, which was subsequently used for automated fiber tracking. In native space, the ROIs did not differ significantly between groups in size as measured by surface area (*t*_(97)_ = 0.222, *P* = 0.825), which could affect the number of dissected streamlines.

**Figure 3.**
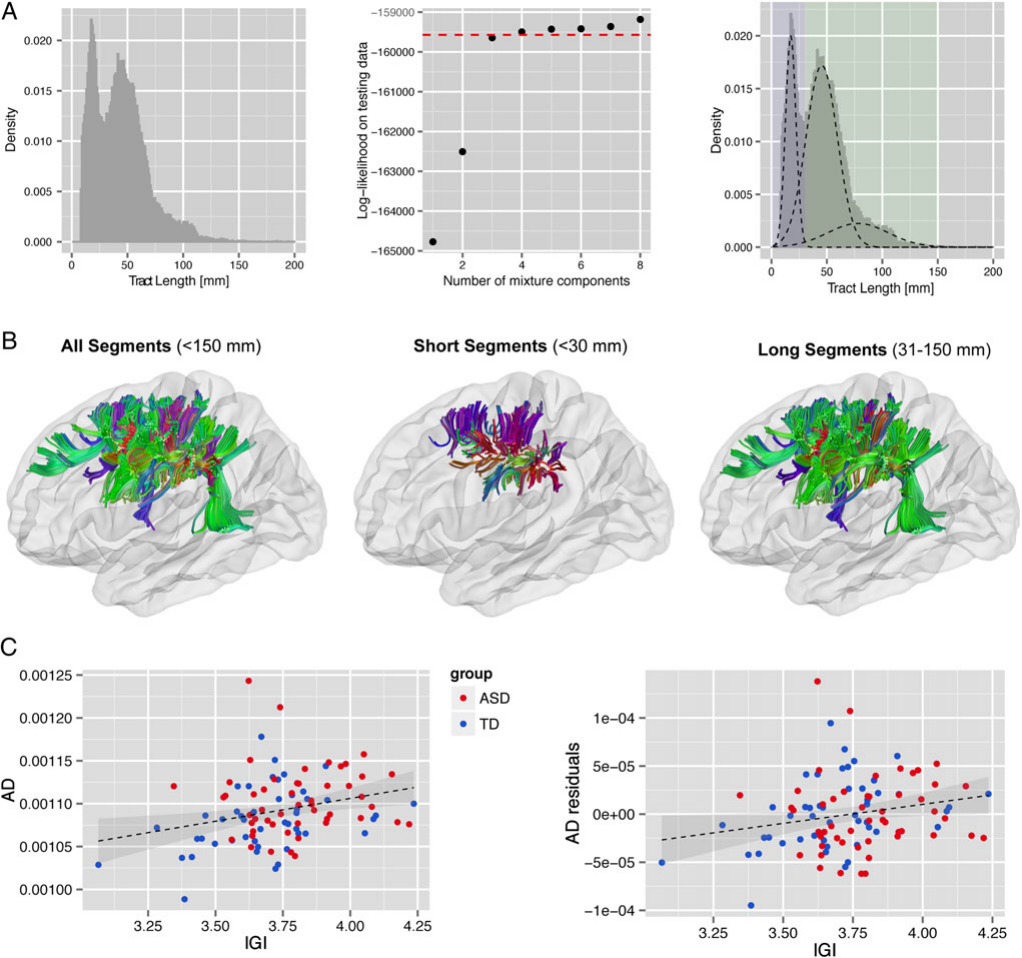
(*A*) Length distribution of tracts originating and/or terminating in the surface-based ROI. The distribution was best modeled using a set of 3 Gaussian Mixture Models, which are indicative of at least 2 dominant tract classes within the cluster. Based on the Mixture Models, a cut-off of 30 mm was utilized to separate short- from long-distance tracts within the cluster (*B*). There was a significant positive correlation between *l*GI measures and AD in short- but not long-distance tracts across groups (left panel) and when controlling for between-group differences (right panel). ADres denotes the AD residuals after the removal of the main effect of group (*C*).

When examining the distribution of tract lengths for all tracts originating and/or terminating in the ROI, we found that the distribution significantly deviated from a unimodal distribution (Hardigan's Dip = 0.002, *P* < 0.001), thus indicating the existence of one (or more) tract classes (see Fig. 3*A* left panel). More specifically, the density distribution of tract lengths was best modeled using a mixture of 3 Gaussian distributions of variable mean and standard deviation, above which no significant improvement in fit was observed (Fig. 3*A* middle and right panel). Approximately 95% of all streamlines were < 90 millimeters (mm), and fell within the first 2 Gaussian components. We therefore subdivided streamlines within the surface-based ROI into 2 dominant tract classes based on cut-offs provided by the Gaussian Mixture Models: short fibers <30 mm, and long fibers between 31–150 mm in lengths (Fig. 3*B*). There were no significant between-group differences in the number of dissected streamlines within and across tract classes overall (*t*_<30_(97) = −1.07, *P* = 0.284, *t*_31-150_(97) = 0.04, *P* = 0.969; *t*_<150_(97) = −0.28, *P* = 0.780), which could affect the examined measures of diffusion.

A similar distribution of tract lengths was observed in the control cluster of the contralateral hemisphere, where no significant difference in *l*GI was found (*t*_(97)_ = 1.52, *P* = 0.130) (see Supplementary Fig. 3).

### Between-group Differences in Tract-specific DTI Measures in Tracts Originating or Terminating in the Surface-based ROI

When controlling for the effect of multicenter image acquisition, measures of MD were significantly increased in individuals with ASD relative to TD controls when all tracts originating and/or terminating in the cluster of increased *l*GI were examined (Table 2). We also found a significant increase in MD measures when separating short- from long-distance tracts originating in the surface-based ROI using a test-wise (uncorrected) error rate of *P* < 0.05. However, none of these effects remained significant when controlling for multiple comparisons. A similar pattern of between-group differences was also observed, when covarying for inter-individual differences in total brain volume or total surface area (see Supplementary Table 1).

**Table 2:** Between-group differences in diffusion measures in tracts and/or terminating in the cluster of significant differences in *l*GI (i.e., ASD>control)

	Subject groups		Significance	
	ASD *(n* = 51)	TD controls (*n* = 48)	*F*(df = 1)	*P* value
Short streamlines <30 mm				
FA	0.36 ± 0.05	0.38 ± 0.04	3.31	0.072
MD	0.79 ± 0.07 (×10^−3^)	0.77 ± 0.06 (×10^−3^)	4.96	0.028*
RD	0.64 ± 0.09 (×10^−3^)	0.61 ± 0.07 (×10^−3^)	3.76	0.055
AD	1.10 ± 0.04 (×10^−3^)	1.08 ± 0.04 (×10^−3^)	8.09	0.005**
Long streamlines between 31 and 150 mm				
FA	0.42 ± 0.06	0.44 ± 0.05	4.00	0.047*
MD	0.78 ± 0.05 (×10^−3^)	0.76 ± 0.05 (×10^−3^)	5.72	0.018*
RD	0.59 ± 0.07 (×10^−3^)	0.57 ± 0.06 (×10^−3^)	4.52	0.036*
AD	1.15 ± 0.04 (×10^−3^)	1.14 ± 0.03 (×10^−3^)	2.16	0.144
All streamlines <150 mm				
FA	0.41 ± 0.06	0.43 ± 0.05	3.73	0.056
MD	0.78 ± 0.05 (×10^−3^)	0.76 ± 0.05 (×10^−3^)	5.62	0.019*
RD	0.60 ± 0.08 (×10^−3^)	0.58 ± 0.06 (×10^−3^)	4.32	0.040*
AD	1.14 ± 0.03 (×10^−3^)	1.13 ± 0.03 (×10^−3^)	3.57	0.061

Note: Data expressed as mean ± standard deviation; *F*(df = *1*) statistic for main effect of group resulting from the multivariate GLM; FA, fractional anisotropy; MD, mean diffusivity (mm^2^ s^−1^); AD, axial diffusivity (mm^2^ s^−1^); RD, radial (perpendicular) diffusivity (mm^2^ s^−1^).

*Significant at *P* < 0.05 (2-tailed).

**Significant following Bonferonni correction for multiple comparisons at *P* < 0.00625 (2-tailed).

Notably, in short-distance streamlines exclusively, we found that measures of AD were significantly increased in ASD relative to controls (*F*_97_ = 8.02, *P* = 0.006) even after correction for multiple comparisons. No significant between-group differences in AD were observed in the long-distance streamlines (see Table 2 for details). There were no significant between-group differences in any of the examined DTI measures in the control ROI (see Supplementary Table 2).

### Relationship Between lGI, Measures of Diffusion, and Diagnostic Category

Lastly, we examined the relationship between *l*GI, AD in short streamlines, and diagnostic category in order to establish whether diffusion properties of the cortical WM were (1) related to variations in cortical GM as measured by *l*GI, or vice versa; (2) a consequence of having ASD (i.e., diagnostic category); or (3) resulting from the combination of both. First, we found that differences in AD were primarily related to variations in mean *l*GI within the cluster of significant between-group difference (*F*_1_ = 4.58, *P* = 0.035), whereas there was neither a significant effect of group (*F*_1_ = 2.46, *P* = 0.120), nor a group-by-*l*GI interaction (*F*_1_ = 2.11, *P* = 0.149) (Fig. 3*C*). Similarly, when predicting measures of *l*GI-by-group, AD, and their interaction, we found that measures of *l*GI were significantly predicted by variations in AD (*F*_1_ = 6.29, *P* = 0.014), and only marginally by group (*F*_1_ = 3.91, *P* = 0.056), or the group-by-diffusivity interaction (*F*_1_ = 3.56, *P* = 0.061).

Using SURE, we also found (1) that the effect of *l*GI on the AD of short tracts significantly exceeded the effect of AD on measures of *l*GI (*χ^2^*(1) = 6.29, *P* = 0.012), and (2) that the effect of group on measures of *l*GI significantly exceeded its effect on measures of AD (*χ^2^*(1) = 3.91, *P* = 0.048). Thus, differences in GM gyrification and WM connectivity are significantly linked. Moreover, measures of cortical gyrification seem more predictive for WM differences than diagnostic status (i.e., having ASD).

The correlation between the *l*GI and AD in short tracts remained significant even when partialling out the effect of mean CT (*r*_par_ = 0.286, *P* = 0.002), sulcal depth (*r*_par_ = 0.274, *P* = 0.003), or radial curvature (*r*_par_ = 0.275, *P* = 0.003) across groups, and when controlling for the effect of these additional cortical features within the GLM. Out of the 3 additional features examined, only mean CT explained significant variability in AD of short tracts in addition to the *l*GI (*F*(1) = 4.44, *P* = 0.003). Thus, WM characteristics are not only dependent on the degree of cortical gyrification, but may also be associated with other morphometric GM features including CT.

## Discussion

This study examined the relationship between GM surface anatomy and characteristics of the underlying WM connectivity in male adults with ASD and TD controls. First, we found that gyrification of the brain in ASD was significantly increased relative to controls in a large left-hemisphere cluster centered around the central sulcus. In this cluster, there was no commensurate increase in vertex-wise estimates of surface area, thus suggesting that these morphometric features represent independent sources of neuroanatomical variability. Using DTI, we subsequently examined between-group differences in WM tracts originating and/or terminating in the surface-based cluster of increased *l*GI in ASD. We found that measures of AD in short but not long tracts were significantly increased in ASD based on their respective distance from the cortical sheet. Furthermore, there was a significant positive relationship between measures of local gyrification and AD in short tracts. This relationship was not significantly modulated by diagnostic status, which was more predictive for GM characteristics than for WM properties, hence implying that ASD might affect WM differences via the cortical GM. Taken together, our findings suggest that gray and white matter abnormalities are closely linked, and may reflect common rather than distinct etiological pathways. However, this relationship is scale-dependent and primarily affects fiber tracts in close proximity to the cortical sheet.

### Atypical Gyrification in ASD

Atypical patterns of gyrification in ASD have been noted previously although findings remain highly variable with regard to the sign and the regional pattern of differences. For example, Hardan et al. (2004), using a manual 2D tracing approach, reported greater prefrontal gyrification of the brain in children and adolescents with ASD relative to controls, while no significant differences were found among adults (Hardan et al. 2004). Previous studies implementing the *l*GI reported significant increases in gyrification in bilateral posterior brain regions in males with ASD compared with TD controls (12–23 years) (Wallace et al. 2013), and significant reductions in *l*GI in the left supramarginal gyrus in males aged 8–40 years (Libero et al. 2014), and in the right inferior frontal and medial parieto-occipital cortices in children with ASD (Schaer et al. 2013). Furthermore, atypical cortical gyrification in ASD has been demonstrated using a variety of alternative metrics, including measures of sulcal morphometry (Levitt et al. 2003), sulcal depth (Nordahl et al. 2007), and gyral complexity (Williams et al. 2012). Our finding of an increased *l*GI in ASD therefore agrees with some—but not all—previous observations in comparable groups of individuals.

While these divergent findings can be partially explained by differences in sample size, demographics and analytical techniques, evidence also suggests that patterns of cortical gyrification are highly variable across individuals, even in normative populations, and that both genetic and nongenetic factors contribute to the formation of cortical gyri (Bartley et al. 1997). For example, Kates et al. (2009) report that cortical folding patterns are highly discordant between monozygotic twins, where one had a diagnosis of ASD, although ASD individuals and their co-twins both exhibited increased parietal-lobe folding relative to unrelated TD controls (Kates et al. 2009). This finding contrasts with conventional volumetric measures of brain anatomy, which are highly concordant between twins, and are therefore largely genetically determined (White et al. 2002). Moreover, cortical folding is not significantly correlated with total brain weight or volume, or with body weight and length, which are under strict genetic control (Zilles et al. 1988; Rogers et al. 2010). Measures of gyrification thus seem to be particularly sensitive to environmental (i.e., nongenetic) factors, and reflect a degree of plasticity that is independent of overall brain size (Zilles et al. 2013). Variability in findings across studies may therefore also be due to the high degree of inter-individual variation that is naturally associated with the *l*GI measure, which directly impacts on statistical effects. Effect sizes rely not only on the mean difference between groups but also on the standard error of measurement associated with a parameter. Differences in variability across samples may therefore influence the regional pattern of between-group differences in *l*GI across studies. This particularly applies to complex neurodevelopmental conditions such as ASD, where genetic effects are expected to interact with environmental factors to give rise to a heterogeneous neurophenotype. On the other hand, the high inter-individual variability associated with the *l*GI makes this measure particularly suited for the investigation of genetic and nongenetic factors driving the atypical development of the brain in ASD, and to disentangle the large phenotypic variability typically observed among ASD individuals.

Neurobiologically, the formation of cortical convolutions has been linked to various cellular mechanisms, although it is likely that there is no singular mechanism that could explain the highly complex patterns of cortical convolutions that are typically observed across the cortex. Most theories relate cortical folding to the neurobiological mechanisms mediating cortical expansion; that is, as the cortex expands it eventually needs to fold to fit an increasing surface area into the restricted space of the skull. For example, the radial unit hypothesis links cortical expansion to the early proliferation of radial unit progenitors, and thus an increase in radial glial cells, which provide scaffold for migrating neurons in the developing cortex (Rakic 1995). Recent studies also suggest that cortical folding may be aided by so-called intermediate radial glia cells that are exclusively found in the gyrencephalic cortex, and that facilitate the tangential—in addition to the radial—dispersion of neurons (Reillo et al. 2011; Wang et al. 2011). In the mature brain, the degree of cortical folding has also been related to (1) the neural complexity of a brain regions in terms of neuronal numbers, synaptic density, and/or dendritic arborization (Welker 1990), and (2) differences in growth rates between the outer and inner cortical layers (Richman et al. 1975; Kriegstein et al. 2006). Thus, the neural mechanisms underlying cortical folding and cortical expansion are intrinsically linked. It is therefore perhaps surprising that we did not observe a commensurate increase in vertex-wise estimates of surface area within the cluster of increased *l*GI, which agrees with a previous report by Wallace (Wallace et al. 2013). Mathematically, however, it is very difficult to compare the 3D nature of the *l*GI with the 2D nature of point-wise estimates of surface (i.e., the average area of triangles/faces touching a vertex). For example, it has previously been shown that the cortex is intrinsically curved, and so cannot be flattened or ‘unfolded’ without having to tear or shear the surface (Griffin 1994; Ronan et al. 2011). This means that the area of a circle with radius *r* on the 3D folded surface of the brain does not directly translate to the area of the same circle in-plane. In ASD, it has also been shown that 3D characteristics of the cortical surface significantly differ from controls, which may impact on the comparability of cortical features between groups. For instance, Ecker et al. (2013) demonstrated that in the cluster where we observed an increase in *l*GI, cortico-cortical separation distances (i.e., shortest path connecting 2 points along the cortical surface) were significantly reduced in ASD (Ecker et al. 2013), indicating that the brain in ASD may be more intrinsically curved. It thus seems that the potential increase in surface area associated with the *l*GI cannot be adequately captured by point- or vertex-wise estimates of surface area, and that both features reflect different sources of neuroanatomical variability.

### Relationship Between lGI and White matter Characteristics

Our study further demonstrates that the GM pathology of ASD, as measured by *l*GI, is closely linked with abnormalities of the underlying WM. First, we found that tracts originating and/or terminating in the cluster of increased *l*GI also have increased axial diffusivity, and this was specific to short tracts <30 mm. Evidence for atypical short-distance tracts in ASD comes from previous DTI studies (e.g., Sundaram et al. 2008; Shukla et al. 2011), which supports the notion that aberrant long-distance connectivity in ASD may be accompanied by intact or even enhanced short-distance connectivity (Casanova et al. 2002; Rubenstein and Merzenich 2003; Belmonte et al. 2004; Courchesne and Pierce 2005). Moreover, our finding agrees with previous reports suggesting that the relationship between GM and WM characteristics may be scale-dependent, with short-distance tracts being more closely associated with GM variability than long-distance tracts (Schaer et al. 2013). However, the definition of short versus long tracts is ultimately arbitrary, and also significantly differs across prior studies. For example, Herbert et al. (2004) subdivided cortical WM based on proximity to the cortical sheet, and this is comparable to our approach (as tracts were separated according to their lengths relative to the surface-based label). While this allows us to further separate tract classes in an automated fashion, it is important to note that the length of a particular tract is not necessarily indicative of its particular composition, even though WM in close proximity to the cortical sheet is likely to contain proportionately more “short” (i.e., cortico-cortical, U-shaped) fibers than “long” (i.e., association or projection) tracts relative to the deeper WM. In future research, it will thus be important to investigate the relationship between GM and WM at different scales, in order to establish the scale at which their association starts to break down.

No significant between-group differences in DTI measures were observed in the contralateral control region, where individuals with ASD did not differ significantly from controls in *l*GI. While the unilaterality of differences in *l*GI is interesting in itself, as it suggests that the trajectory of brain development is idiosyncratic for different hemispheres in ASD, there is currently no sufficient evidence that could explain our finding. Notably, many of the functional impairments typically associated with ASD (e.g., language and fine motor skills) are associated with a left hemispheric specialization (reviewed in Floris et al. 2016). Moreover, it has previously been shown that the left hemisphere neuroanatomy is more predictive of ASD diagnosis than the right hemisphere (Ecker et al. 2010). It will therefore be important in the future to establish whether hemispheric differences in *l*GI are accompanied by hemispheric differences in neurodevelopmental trajectories, and how these relate to autistic symptoms.

While it may be tempting to speculate about the underlying pathological substrate of WM diffusivity, it is however very difficult to unequivocally associate them with specific biophysical changes, and particularly measures of directional diffusivity. Some evidence suggests that radial diffusivity (i.e., the diffusion of water perpendicular to WM fibers) is related to the degree of myelination (Song et al. 2002; 2005), while decreased AD (i.e., diffusion of water molecules parallel to fibers) is indicative of axonal damage (Song et al. 2003). For example, AD is highly correlated with axonal damage in mice with autoimmune encephalomyelitis (Budde et al. 2009), and mouse models of other WM injuries (DeBoy et al. 2007). This would imply that our finding of increased AD in individuals with ASD may be driven by differences in axonal characteristics rather than myelination. The interpretation of the DTI measures examined in our study is further complicated by several confounding factors, including the degree of cerebrospinal fluid partial volume effects (Vos et al. 2011), and crossing fibers (Wheeler-Kingshott and Cercignani 2009). There are also intrinsic tractography biases that may affect the comparison of short and long fibers; for example, longer paths are less likely to be reconstructed than shorter paths due to accumulated error, and curved paths are less likely to be reconstructed than straighter paths due to the eigenvector pointing “away” from the tract (Girard et al. 2014). Thus, while our finding provides some support for atypical short-distance WM connectivity in ASD, future histological studies are needed to better characterize the specific microstructural determinants of WM abnormalities in ASD.

Last, we found that there was a significant positive relationship between the *l*GI and AD in short tracts, which suggests that variations in GM neuroanatomy and WM connectivity are closely linked. Notably, the relationship was not significantly modulated by diagnostic status, which was more predictive for GM characteristics than for WM properties, implying that ASD might affect WM differences via the cortical GM. Our finding agrees with genetic studies highlighting the important role of genes affecting GM development in the etiology of ASD. For example, many of the common underlying molecular pathways implicated in ASD include genes involved in cell proliferation and neuron motility, cell adhesion and axon targeting, and synaptogenesis and synapse differentiation (e.g., Betancur et al. 2009; Pinto et al. 2010; Gilman et al. 2011). Perturbations to the genetic and molecular mechanisms underlying the typical development of the micro-circuitry of the brain are thus likely to also affect the development of the brain's WM macro-circuitry. On the other hand, there is less evidence for an involvement of genes that target myelination directly. For example, according to the SFARI Gene List (https://gene.sfari.org/), only 10 out of 706 risk genes for ASD are involved in myelination, suggesting that the genetic load for aberrant gray matter and axonal connectivity in ASD may be higher than for atypical myelination. However, due to the cross-sectional nature of the study in an adult population, our results naturally do not allow us to disentangle the causal mechanisms that drive the association between the *l*GI and WM diffusivity. While some of our results indicate that the *l*GI predicts WM diffusivity significantly better than vice versa, we cannot exclude the possibility that the association is driven by a third mechanism affecting both gray and white matter neuroanatomy. Furthermore, we found that mean CT within the cluster significantly predicted measures of AD in addition to the *l*GI. Cortical gyrification is therefore not the only GM feature that predicts WM connectivity. Taken together, while our study demonstrates that gray and white matter abnormalities are closely linked during adulthood, and may reflect common rather than distinct etiological pathways, future longitudinal studies are needed to elucidate the causative link between atypical gray and white matter development in ASD.

### Methodological Limitations

The current study has a number of additional limitations. First, a multicenter design was used for MRI data acquisition to overcome single-site recruitment limitations. However, we utilized a standardized protocol for multicenter acquisition (Deoni et al. 2008), and also accounted for inter-site effects in the statistical model (Suckling et al. 2014). Our findings are therefore unlikely to be driven by variance components unrelated to the between-group difference. Second, while automated tractography allows for the investigation of a large number of tracts in a large sample of individuals, our approach relies on the accurate coregistration of surface reconstructions and DTI volumes. We, however, did not find any significant differences in the number of streamlines within and across tract classes between individuals with ASD and controls, and the volumetric ROIs also did not differ significantly between groups in size. Moreover, no significant between-group differences were observed in the contralateral hemisphere, which served as a control ROI. While this does not exclude the possibility of registration errors, it seems that there was no systematic registration bias that could affect the size and number of examined streamlines across groups. In the future, it will also be crucial to address the important issue of crossing fibers (e.g., using a deconvolution instead of conventional tensor models; Dell'acqua et al. 2013) and partial volume effects, which might affect the accuracy of estimated DTI metrics. Furthermore, the benefit of using alternative (e.g., non-linear) approaches for image (or surface) registration may be explored in the future in order to reduce inter-individual variability in DTI measures. However, all of these issues are expected to affect both ASD and TD group to an equal degree, and between-group comparisons are thus unlikely to be affected by these confounds. Lastly, it is important to note that ASD is a disorder that recruits multiple neural systems, which—as a whole—mediate the cluster of clinical symptoms characteristic for ASD. Thus, while our approach based on differences in *l*GI captures some aspects of perturbed brain connectivity in ASD, it is by no means sufficient to explain all atypical connections. Further research will be required to determine if our findings generalize to other neuro-cognitive systems that are affected in ASD.

## Supplementary Material

Supplementary Material can be found at *http://www.cercor.oxfordjournals.org/* online.

## Funding

This work was supported by the Autism Imaging Multicentre Study Consortium funded by Medical Research Council UK Grant (G0400061), by the EU-AIMS Consortium receiving support from the Innovative Medicines Initiative Joint Undertaking under grant agreement no. 115300, which includes financial contributions from the European Union Seventh Framework Programme (FP7/2007-2013), from the European Federation of Pharmaceutical Industries and Associations companies in kind, and from Autism Speaks. Funding to pay the Open Access publication charges for this article was provided by MRC.

## Appendix

The Medical Research Council Autism Imaging Multicentre Study Consortium (MRC AIMS Consortium) is a UK collaboration between the Institute of Psychiatry, Psychology and Neuroscience (IoPPN) at King's College, London, the Autism Research Centre, University of Cambridge, and the Autism Research Group, University of Oxford. The Consortium members are in alphabetical order: Anthony J. Bailey (Oxford), Simon Baron-Cohen (Cambridge), Patrick F. Bolton (IoPPN), Edward T. Bullmore (Cambridge), Sarah Carrington (Oxford), Marco Catani (IoPPN), Bhismadev Chakrabarti (Cambridge), Michael C. Craig (IoPPN), Eileen M. Daly (IoPPN), Sean C.L. Deoni (IoPPN), Christine Ecker (IoPPN), Francesca Happé (IoPPN), Julian Henty (Cambridge), Peter Jezzard (Oxford), Patrick Johnston (IoPPN), Derek K. Jones (IoPPN), Meng-Chuan Lai (Cambridge), Michael V. Lombardo (Cambridge), Anya Madden (IoPPN), Diane Mullins (IoPPN), Clodagh M. Murphy (IoPPN), Declan G.M. Murphy (IoPPN), Greg Pasco (Cambridge), Amber N.V. Ruigrok (Cambridge), Susan A. Sadek (Cambridge), Debbie Spain (IoPPN), Rose Stewart (Oxford), John Suckling (Cambridge), Sally J. Wheelwright (Cambridge), Steven C. Williams (IoPPN), and C. Ellie Wilson (IoPPN).
